# CD44 Is Associated with the Aggressive Phenotype of Nasopharyngeal Carcinoma through Redox Regulation

**DOI:** 10.3390/ijms140713266

**Published:** 2013-06-26

**Authors:** Chien-Hung Lin, Peir-Haur Hung, Yann-Jang Chen

**Affiliations:** 1 Institute of Clinical Medicine, National Yang-Ming University, Taipei 112, Taiwan; E-Mail: dan08@tpech.gov.tw; 2 Department of Pediatrics, Zhongxing Branch, Taipei City Hospital, Taipei 103, Taiwan; 3 Department of Medical Research, Ditmanson Medical Foundation Chia-yi Christian Hospital, Chia-yi 600, Taiwan; E-Mail: dtmedg3@yahoo.com.tw; 4 Department of Life Sciences and Institute of Genome Sciences, National Yang-Ming University, Taipei 112, Taiwan; 5 Department of Pediatrics, Renai Branch, Taipei City Hospital, Taipei 106, Taiwan

**Keywords:** CD44, nasopharyngeal carcinoma, cancer stem cells, epithelial-mesenchymal transition, reactive oxygen species

## Abstract

Recent studies have shown that cancer stem-like cells (CSCs) within a tumor have the capacity for self-renewal and differentiation, and are associated with an aggressive phenotype and therapeutic resistance. Studies have also associated tumor progression with alterations in the levels of intracellular reactive oxygen species (ROS). In this study, we cultured nasopharyngeal carcinoma (NPC) CSCs in conditions that allowed sphere formation. The resulting sphere cells displayed stemness properties, characteristics of the epithelial–mesenchymal transition (EMT), and increased expression of the CSC surface marker CD44. We further evaluated the association between CD44 expression and EMT marker expression, and any correlation with redox status, in these CSCs. We showed that the EMT in sphere cells is associated with the upregulation of CD44 expression and increased ROS generation, which might promote NPC aggressiveness. We also identified the coexpression of CD44 with the EMT marker N-cadherin in sphere cells, and downregulated CD44 expression after the addition of the antioxidant N-acetyl cysteine. Our results indicate that CD44 plays a role in the EMT phenotype of CSCs in NPC, and suggest its involvement in EMT-associated ROS production. These findings might facilitate the development of a novel therapy for the prevention of NPC recurrence and metastasis.

## 1. Introduction

Nasopharyngeal carcinoma (NPC) is a distinctive type of head and neck cancer that is highly prevalent in southeast China and Taiwan. Previous epidemiologic and experimental studies have shown the close association between EBV infection and the initiation of NPC [1]. Unlike other head and neck cancers, NPC is associated with a high tendency for invasion of the surrounding tissues and metastasis to regional lymph nodes at an early stage. Mortality in NPC patients is typically caused by local recurrence and distant metastases [2]. Although radiotherapy, or a combination of radiotherapy and chemotherapy, are traditional treatment modalities for NPC, a considerable percentage of patients with advanced NPC suffer from relapse or metastatic diseases. Therefore, to effectively develop targeted molecular therapies, the underlying mechanisms causing NPC recurrence and metastasis must be elucidated.

Recent research has provided evidence to show that cancer stem-like cells (CSCs), or tumor-initiating cells, have unique characteristics such as the ability for self-renewal and to generate differentiated cells. CSCs play a key role in tumor resistance to chemotherapy and radiotherapy. During metastasis, CSCs often undergo an epithelial–mesenchymal transition (EMT), which results in a mesenchymal fibroblast-like morphology, reduced intercellular adhesion, increased motility, and increased invasive and migratory properties [3]. The EMT-type tumor cells display several of the characteristics of CSCs, and are closely associated with tumor recurrence and therapeutic resistance [4].

Previous studies on nasopharyngeal tumors identified CD44 as a marker of CSCs, indicating its potential use as a therapeutic target [5,6]. Further investigation of the role of CD44 in NPC progression could thus potentially facilitate the development of an effective NPC treatment. Studies have also associated the aggressive and invasive behaviors of tumor cells with increased levels of intracellular reactive oxygen species (ROS), resulting in malignant phenotypes and metastatic outcomes [7,8]. Adaptation to oxidative stress is crucial for CSC survival, and previous experiments have shown these cells to display intracellular antioxidant capacities and increased radiochemotherapeutic resistance [9,10]. Several studies have shown ROS to participate in the EMT in several tumor types, indicating their involvement in tumor metastasis and changes in the microenvironment [11,12]. However, few studies have evaluated the association between ROS generation and the EMT in CSCs.

The purpose of this study was to evaluate the potential association between the EMT and increased CD44 expression in NPC, and to identify the possible role of CD44 in the regulation of intracellular ROS levels and the promotion of the NPC aggressive phenotype.

## 2. Results

### 2.1. NPC Sphere-Derived Cells Show Stem-Like Properties and Chemotherapeutic Resistance, and Express the Mesenchymal Phenotype

**Figure 1:**
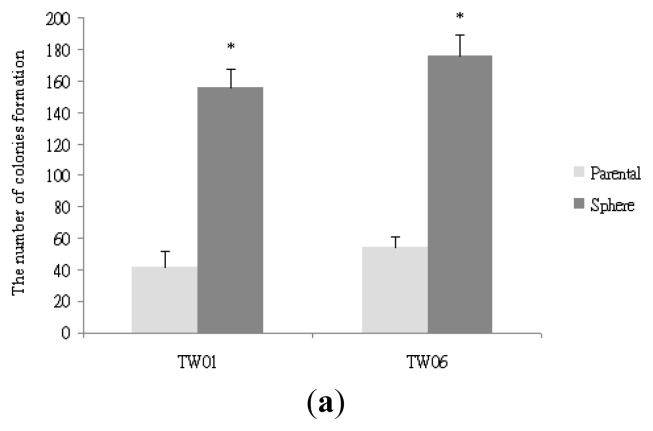

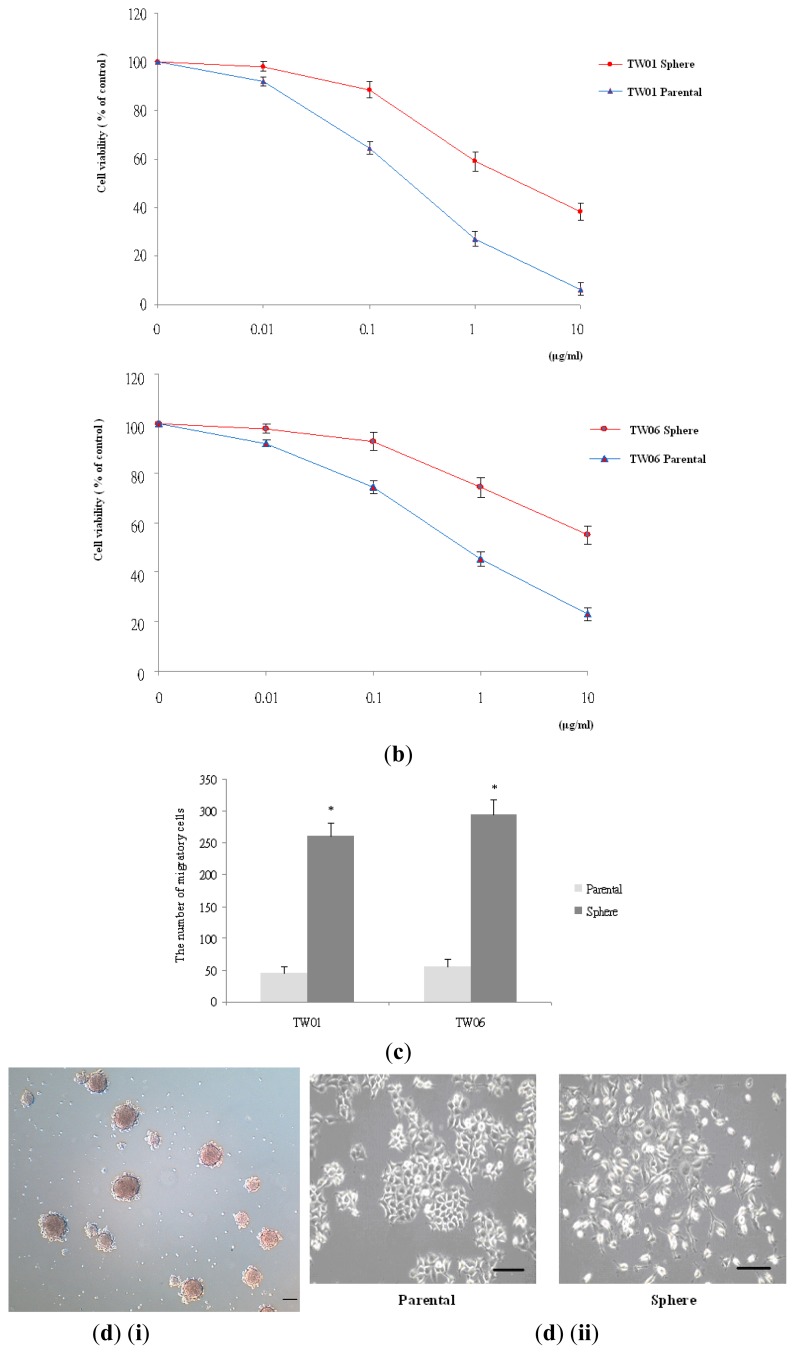

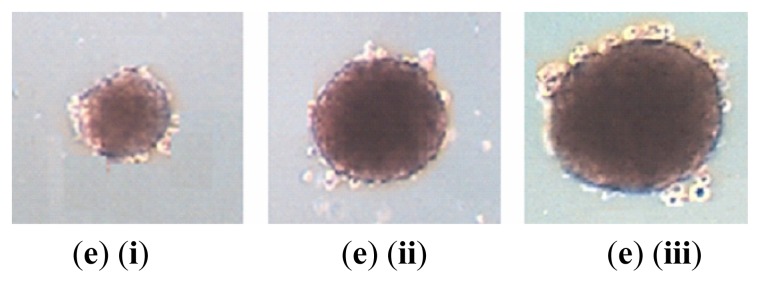
Characterization of NPC sphere-derived cells cultured anchorage-independently. (**a**) The NPC sphere-derived cells showed significantly higher colony-forming ability than the parental cells, * *p* < 0.01; (**b**) The NPC sphere-derived cells showed higher survival rates than the parental cells after cisplatin treatment; (**c**) The NPC sphere-derived cells showed more potent invasive behavior than the parental cells, * *p* < 0.01; (**d**) Distinct properties of the NPC sphere-derived cells and parental cells. (**i**) TW06 sphere cells cultured nonadherently in a serum-free medium; (**ii**) The differing morphologies of the TW06 parental and sphere-derived cells when cultured adherently in a 10% serum for 48 h (scale bar 100 μm); (**e**) Serial photographs during non-adherent culture demonstrated sphere growing progressively at 4 days (**i**), 8 days (**ii**), and 12 days (**iii**).

To investigate the tumorigenicity of NPC cells according to colony formation ability, we performed a soft agar assay on TW01 and TW06 parental and sphere-derived cells. As shown in Figure 1a, our results showed that the NPC sphere cells grown anchorage-independently had significantly higher colony-forming ability than the parental cells. These data indicated that the sphere-derived cells exhibited more potent tumorigenicity than the parental cells *in vitro*. We then evaluated cell proliferation by using the MTT assay to determine the chemoresistant properties of the self-renewing sphere-derived cells. As shown in Figure 1b, the sphere-derived cells showed higher survival rates than the parental cells after cisplatin treatment, suggesting their involvement in properties of therapeutic resistance and aggressive behavior in NPC. As shown in Figure 1c, we then evaluated the invasive capacity of the NPC parental and sphere-derived cells and identified higher invasive ability in the sphere-derived cells than in the parental cells. Figure 1d shows that the NPC cells cultured anchorage-independently displayed a spindle-like morphology when attached in the monolayer. As shown in Figure 1e, when sphere cells were dissociated into single cells, they were found capable to generate new spheres and increased progressively in diameter during culture, suggesting that NPC sphere cells have capacity for self-renewal.

### 2.2. Stemness Gene, Drug-Resistant Gene, and EMT Marker Expression in NPC Sphere-Derived Cells

**Figure 2:**
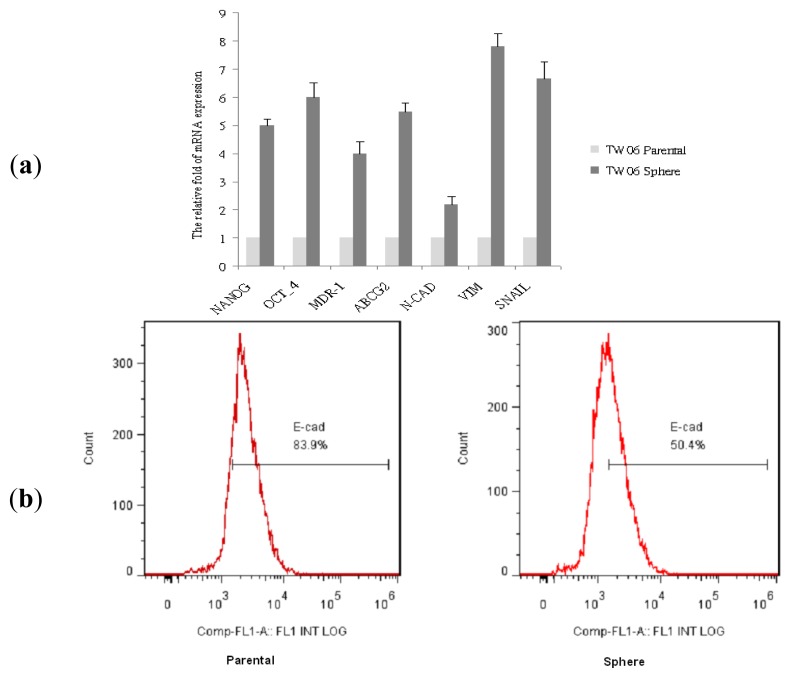
Stemness gene, drug-resistant gene, and EMT marker expression in sphere-derived cells. The expression of (**a**) stemness gene (Oct-4 and Nanog), drug-resistant gene (MDR and ABCG2), and EMT marker (N-cad, vimentin, and snail) mRNA was significantly higher in the TW06 sphere-derived cells than in the parental cells; (**b**) The TW06 sphere-derived cells displayed lower E-cad expression than the parental cells do.

Analyses of gene expression using the qRT-PCR indicated that the expression of the stemness genes Oct-4 and Nanog, and the expression of the drug-resistant genes MDR and ABCG2, was significantly higher in the sphere-derived cells than in the parental cells. As shown in Figure 2a, the expression of the mesenchymal markers *N*-cadherin (*N*-cad), vimentin, and snail was also significantly higher in the sphere-derived cells than in the parental cells. The flow cytometric analysis data in Figure 2b showed lower expression of the epithelial marker E-cadherin (E-cad) in the sphere-derived cells compared with the parental cells. These results indicated that the NPC sphere-derived cells have more potent tumorigenic and invasive ability than the parental cells do, and show high stemness characteristics, drug resistance, and EMT marker expression, suggesting the acquisition of aggressive phenotype.

### 2.3. EMT Marker Expression Is Associated with CD44 Expression in NPC Sphere-Derived Cells

**Figure 3:**
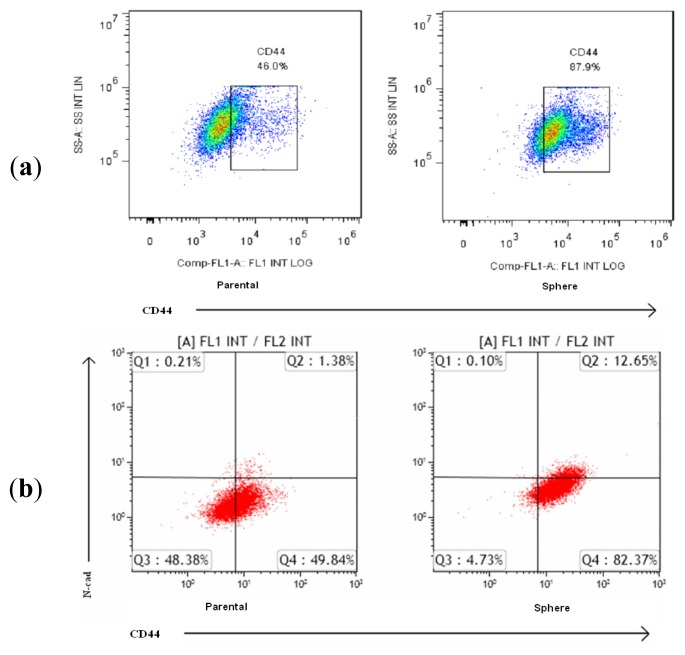
EMT marker expression in NPC sphere-derived cells is associated with increased surface marker CD44 expression. (**a**) The TW06 sphere-derived cells displayed higher CD44 expression than the parental cells did; (**b**) Flow cytometric analysis of CD44 and *N*-cad expression in the TW06 sphere-derived cells and parental cells.

Previous studies have shown that CD44-positive cells play a critical role in the CSCs of NPC. Our data indicated that NPC cells display stem-like properties, EMT changes, and increased CD44 expression when cultured anchorage-independently. As shown in Figure 3a, CD44 expression was significantly higher in the sphere-derived cells than in the parental cells. As shown in Figure 3b, flow cytometric analysis with double-staining showed significantly higher expression of the mesenchymal marker N-cad and CD44 in the sphere-derived cells than in the parental cells.

### 2.4. Redox Status in NPC Sphere-Derived Cells Is Associated with CD44 and EMT Marker Expression

**Figure 4:**
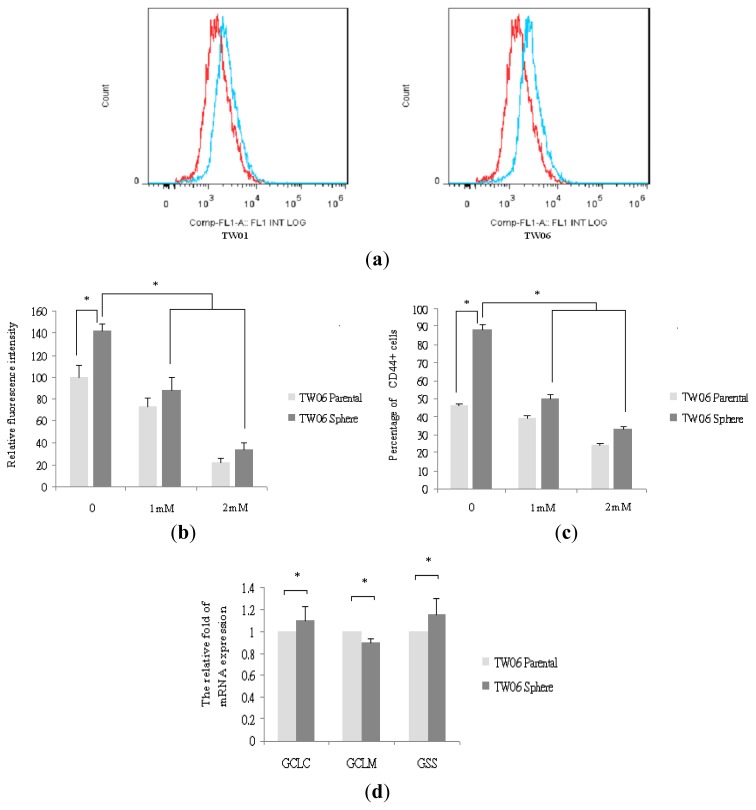

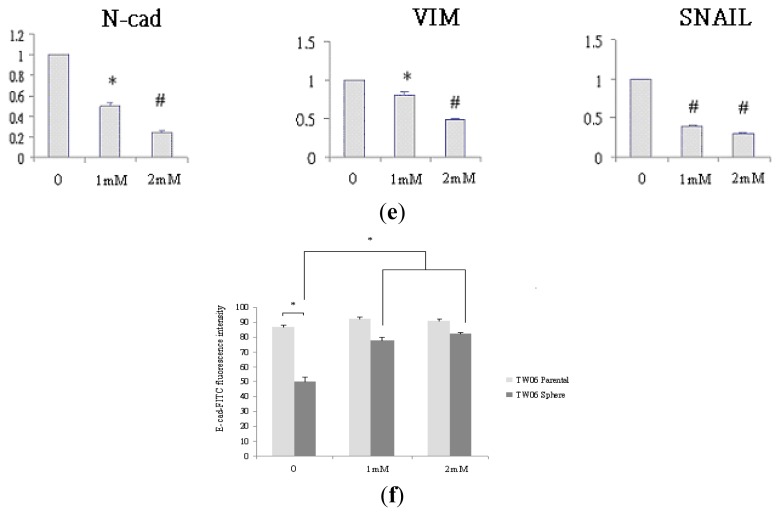
Redox status in NPC sphere-derived cells is associated with CD44 and EMT marker expression. (**a**) The levels of ROS were higher in the sphere-derived cells than in the parental cells. Red, parental cells; blue, sphere-derived cells; (**b**) The levels of ROS reduced significantly in the TW06 sphere-derived cells after the addition of NAC, as shown by DCF-DA staining, * *p* < 0.05; (**c**) The expression of CD44 reduced significantly in the TW06 sphere-derived cells after the addition of NAC, * *p* < 0.05; (**d**) The expression of GCLC, GCLM, and GSS mRNA showed non-significant differences in the TW06 sphere-derived cells and parental cells, * *p* > 0.05; (**e**) NAC treatment reduced the levels of N-cad, VIM, and snail mRNA in the TW06 sphere-derived cells. Data are presented as mean ± SD. * and # indicate significant differences from the respective controls, *p* < 0.05; (**f**) NAC treatment increased the expression of E-cad in the TW06 sphere-derived cells, * *p* < 0.05.

To evaluate the changes in redox status during the EMT in NPC, we measured intracellular ROS levels using prooxidants and DCF-DA staining. As shown in Figure 4a, our data indicated higher ROS levels in the sphere-derived cells than in the parental cells, suggesting that the EMT process is associated with ROS generation. As shown in Figure 4b,c, to confirm the involvement of the ROS in the EMT, we treated the sphere-derived cells with the antioxidant NAC in various concentrations, and observed reduced ROS levels and reduced CD44 expression after NAC treatment.

As shown in Figure 4d, our qRT-PCR data indicated that GCLC, GCLM, and GSS mRNA expression showed non-significant differences in the sphere-derived cells and parental cells. These results suggested that the levels of ROS during the EMT are mediated by CD44 rather than the ROS scavengers GCLC, GCLM, and GSS. As shown in Figure 4e, NAC treatment reduced *N*-cad, vimentin, and snail mRNA expression in the sphere-derived cells. In contrast, and as shown in Figure 4f, NAC treatment increased E-cad expression in the sphere-derived cells. These findings indicate that CD44 plays a pivotal role in the EMT properties of NPC CSC, which might contribute to ROS generation and aggressive behavior.

**Figure 5:**
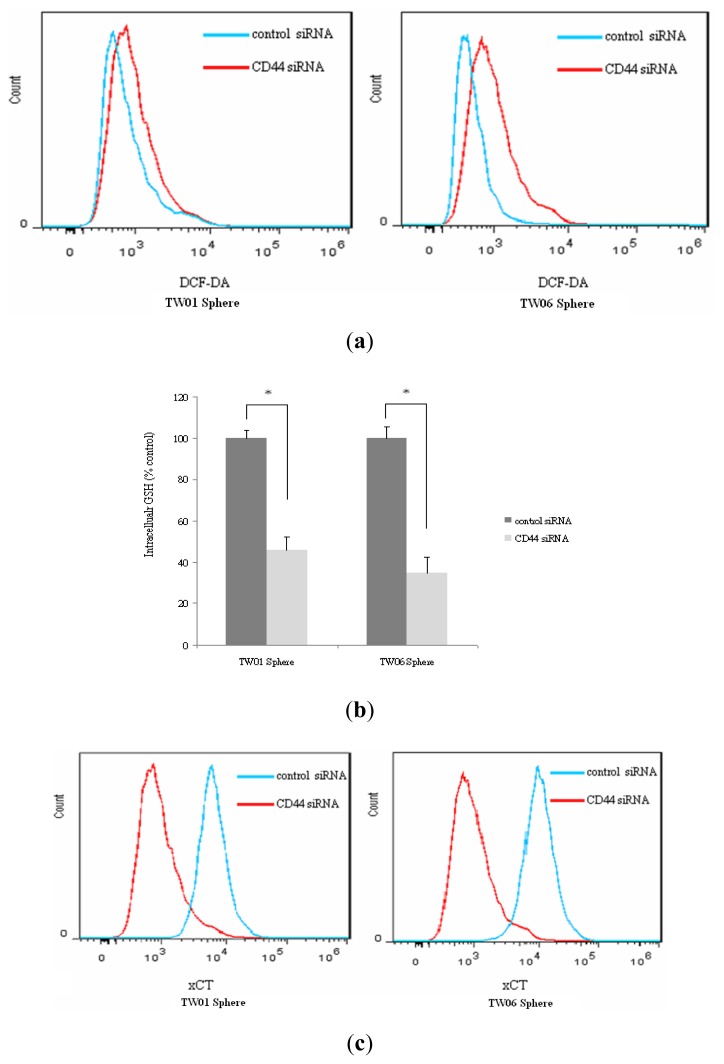
CD44 correlates with ROS defense, intracellular GSH level and cell surface xCT expression in NPC sphere cells. (**a**) Flow cytometric analysis of ROS level after CD44 knockdown by control and CD44 siRNA; (**b**) Intracellular GSH level of sphere cells was reduced after transfected with CD44siRNA. * *p* < 0.05; (**c**) Expression of cell surface xCT was analyzed after CD44 knockdown by control and CD44 siRNA.

To further confirm the role of CD44 in ROS defense in NPC sphere cells, knockdown of CD44 using a small interfering RNA (siRNA) was performed. As shown in Figure 5a, the flow cytometric analysis showed an increase in DCF-DA staining in sphere cells by transfection with CD44 siRNA compared with those transfected with control siRNA. In addition, Figure 5b shows that the amount of GSH in sphere cells transfected with CD44 siRNA was found to be significantly lower than that in those transfected with control siRNA, suggesting that CD44 contributes to enhancement of the intracellular GSH synthesis. To investigate whether the expression of the cystine transporter subunit (xCT) is related to CD44 in NPC cells, we examined the amount of xCT at the surface of sphere cells by flow cytometry. As shown in Figure 5c, the level of xCT at the surface of sphere cells transfected with control siRNA was obviously higher than on sphere cells transfected with CD44 siRNA, suggesting that cell surface expression of xCT is related to CD44. All these findings provide clues that CD44 plays a role in the regulation of ROS defense and tumor progression.

## 3. Discussion

Previous studies have shown that CSCs are associated with tumor initiation and characteristics of self-renewal and differentiation. They also reportedly play an essential role in the generation of therapeutic resistance. CSCs undergoing metastasis often undergo the EMT, which is a critical event for the induction of changes in morphology and cell motility, and high EMT marker expression is associated with higher epithelial tumor invasiveness and aggressiveness [13,14]. The aberrant expression of EMT markers in NPC is closely associated with lymph node metastasis and an advanced clinical stage. In addition, in NPC patients, high EMT marker expression is predictive of a poor prognostic outcome [15]. Although chemotherapy and radiotherapy can improve the survival rates of NPC patients, the prognosis remains poor in a considerable percentage of patients with relapse or metastatic diseases [16].

In this study, we identified and isolated CSCs from cancer cells using several techniques. Previous studies have shown that CSCs cultured in a suspension exhibiting resistance to anoikis have the ability to form spheres, and that these spheres can express putative stem cell markers and show chemoradiation resistance [17,18]. In our study, we showed that NPC sphere-derived cells growing nonadherently possess CSC properties, including upregulated expression of stemness (Oct-4 and Nanog) and drug-resistant (MDR-1 and ABCG2) genes compared with the monolayer parental cells. We also showed that the EMT is associated with the downregulation of the epithelial marker, E-cad and the upregulation of the mesenchymal markers, snail, vimentin, and N-cad.

Although the self-renewal ability, EMT, metastatic capability, and therapeutic resistance of CSCs have yet to be fully elucidated, the results of our study show that NPC sphere-derived cells that have undergone EMT display increased CD44 expression, indicating the functional relevance of the surface marker in the EMT signature. Accumulating evidence supports that the EMT plays a critical role in tumor invasion and metastasis, and is significantly involved in chemotherapeutic and radiation resistance [19,20].

Our results also demonstrated that NPC cells growing nonadherently in a serum-free medium can be induced to express the EMT phenotype. This process elicited an increase in intracellular ROS that can be reverted by adding an antioxidant (NAC). We observed a higher degree of ROS generation in the sphere-derived cells than in the parental cells, suggesting that the migratory and invasive phenotypes confer high levels of oxidative stress on cancer cells. Consistent with previous studies, the sphere cells’ ROS levels were associated with tumor progression through tumor cell proliferation, survival, migration, and invasion [21,22]. Our study results indicate that the adaption of CSCs undergoing the EMT to a relatively high level of intracellular ROS, is mediated by CD44. Therefore, CD44 might play a crucial role in the regulation of intracellular ROS, thus contributing to metastasis and drug resistance in tumor cells [23,24].

The finding that the scavenging of ROS by CSCs was associated with the inhibition of the EMT provided further convincing evidence of the involvement of ROS generation in the EMT process, and suggested that agents that potently antagonize ROS could have potential use in the prevention of tumor progression and metastasis by reversing the EMT. Previous studies have shown that the potent ROS inhibitor NAC limits cancer cell invasiveness dose-dependently, and that natural antioxidant compounds effectively inhibit cancer initiation in epithelial cells [25,26].

We further found that the NPC sphere-derived cells displayed CSC properties and increased CD44 expression, which is consistent with previous studies’ observations of CSC-like properties in CD44-positive cells in NPC [27,28]. CD44 is a cell surface proteoglycan and glycoprotein that plays a role in cell-matrix interactions and is the principle receptor for targeted cancer therapy [29]. In previous studies, overexpression of CD44 was associated with tumor invasion, metastasis, and drug resistance [30–33]. CD44 is considered the surface marker of a variety of cancers. It can respond to various oxidative stress levels and promotes anchorage-independent growth, migration, and multidrug resistance [34]. Recent findings indicate that a CD44 variant enhances ROS defense in cancer cells through interaction with and stabilization of xCT, which is the cell surface cystine transporter subunit, thereby promoting tumor growth [35,36]. CD44 is also involved in the regulation of the glycolytic pathway by modulating cellular reduced glutathione and contributes to antioxidant status and drug resistance in cancer cells [37]. In addition, several tumor-specific CD44 variants have been described in previous reports [38,39]; further study is necessary to determine which CD44 variant is expressed in NPC.

Our study findings confirmed that the NPC cells undergoing EMT and parental cells display differing CD44 expression levels. We also identified that the changes in CD44 positivity in sphere-derived cells are in accordance with ROS levels, indicating the involvement of CD44 in the modulation of ROS status during the EMT in tumor cells. Our results also showed that reducing oxidative stress can contribute to the downregulation of NPC surface marker expression and the suppression of the EMT, suggesting that the supplementation of ROS-attenuating agents might provide a beneficial strategy for repressing CSC properties and ameliorating the aggressive NPC phenotype. Further studies evaluating the adjuvant benefits of antioxidants are warranted.

## 4. Materials and Methods

### 4.1. Cell Culture

#### 4.1.1. Parental Monolayer Cell Culture

NPC TW01 (WHO type I, keratinizing squamous cell carcinoma) and TW06 (WHO type III, undifferentiated carcinoma) cell lines were established and cultured in 10 cm^2^ dishes by using a Dulbecco’s Modified Eagle Medium (DMEM, Invitrogen Carlsbad, CA, USA), a 10% fetal bovine serum (FBS, BIOIND, Kibbutz Beit Haemek, Israel), 1% sodium pyruvate (BIOIND, Haemek, Israel), 1% penicillin, streptomycin, amphotericin (PSA, BIOIND), and 1% nonessential amino acids (NEAA, BIOIND). The cells were incubated at 37 °C in a humidified atmosphere containing 5% CO_2_.

#### 4.1.2. Nonadherent Culture

The TW01 and TW06 parental cells were seeded nonadhesively in 6-well culture dishes coated with thin agarose at a density of 2 × 10^4^/mm^3^ in a serum-free DMEM/F12 medium (Invitrogen). The culture medium was changed on alternate days until spheres formed. After 7–10 days, the spheres were collected by filtration through a 70 μm mesh used in subsequent experiments.

### 4.2. Soft Agar Clonogenic Assay

The bottom of each well (35 mm) of the 6-well culture dishes was coated with 2 mL of the agar mixture (DMEM, 10% (*v*/*v*) FCS, 0.6% (*w*/*v*) agar). After the solidification of the bottom layer, 2 mL of the top agar-medium mixture (DMEM, 10% (*v*/*v*) FCS, 0.3% (*w*/*v*) agar) containing 2 × 10^4^ parental and sphere-derived cells was added and incubated at 37 °C for 2 weeks. At the end of the incubation period, the number of colonies was counted using a microscope after staining with crystal violet.

### 4.3. Evaluation of Cell Viability Using the 3-(4,5-dimethylthiazol-2-yl)-2,5-diphenyltetrazolium Bromide (MTT) Assay

The effects of cisplatin on proliferating NPC TW01 and TW06 parental and sphere-derived cells were evaluated using the MTT assay. Cells (2.5 × 10^3^ per well) were seeded in 96-well plates and cultured for 24 h. Then, cisplatin or DMSO (the vehicle control) was added after plating onto adherent cells at specified concentrations. Each treatment was performed in triplicate. After 72 h, 20 μL of the MTT solution (5 mg/mL; Sigma, St. Louis, MO, USA) was added to each well and incubated for 4 h at 37 °C. The MTT formazan crystal was then dissolved in the DMSO and the absorbance was measured using a microplate reader (Bio-Rad 680, Bio-Rad Laboratories, Hercules, CA, USA) at a wavelength of 570 nm.

### 4.4. Cell Invasion Analysis

A transwell system with a polycarbonate filter membrane (24-well insert, pore size 8 μm, Corning Costar) was used. Each well was coated with Matrigel (60 μg; BD Bioscience) immediately prior to the invasion assay. The cells were plated in a medium without serum or growth factors, and the medium supplemented with serum was used as a chemoattractant in the lower chamber. The cells were incubated for 48 h, and the cells on the lower surface of the membrane were fixed with methanol and stained with crystal violet. The cells invading the membrane were counted under a light microscope (40×, 3 random fields per well).

### 4.5. Evaluation of Gene Expression using the Quantitative Real-Time Polymerase Chain Reaction (qRT-PCR)

The total RNA was isolated using a Trizol reagent (Invitrogen). First-strand cDNA was reverse transcribed with SuperScript III (Invitrogen). Gene expression of stemness (Nanog, Oct-4), chemo-resistant (MDR-1, ABCG2), mesenchymal (N-Cad Vim, Snail), glutathione-synthesis (Gss, GCLc, GCLm) were analyzed. The primer sequences used for the qRT-PCR were as follows:

GAPDH, Forward: 5′-ACGGGAAGCTCACTGGCATGG-3′; Reverse: 5′-GGTCCACCACCCTGTTGCTGTA-3′
Nanog, Forward: 5′-ATTCAGGACAGCCCTGATTCTTC-3′; Reverse: 5′-TTTTTGCGACACTCTTCTCTGC-3′
Oct-4, Forward: 5′-GTGGAGAGCAACTCCGATG-3′; Reverse: 5′-TGCTCCAGCTTCTCCTTCTC-3′
MDR-1, Forward: 5′-TGGCAAAGAAATAAAGCGACTGA-3′; Reverse: 5′-CAGGATGGGCTCCTGGG-3′
ABCG2, Forward: 5′-CATGTACTGGCGAAGAATATTTGGT-3′; Reverse: 5′-CACGTGATTCTTCCACAAGCC-3′
N-Cad, Forward: 5′-AGGGTGGACGTCATTGTAGC-3′; Reverse: 5′-CTGTTGGGGTCTGTCAGGAT-3′
Vim, Forward: 5′-GAGAACTTTGCCGTTGAAGC-3′; Reverse: 5′-GCTTCCTGTAGGTGGCAATC-3′
Snail, Forward: 5′-CTTCCAGCAGCCCTACGAC-3′; Reverse: 5′-CGGTGGGGTTGAGGATCT-3′
Gss, Forward: 5′-CCTGCTAGTGGATGCTGTCA-3′; Reverse: 5′-TCATCCTGTTTGATGGTGCT-3′
GCLc, Forward: 5′-GTCTTCAGGTGACATTCCAAGC-3′; Reverse: 5′-TGTTCTTCAGGGGCTCCAGTC-3′
GCLm, Forward: 5′-CTGCTAAACTGTTCATTGTAGG-3′; Reverse: 5′-CTATGGGTTTTACCTGTG-3′

GAPDH was used as the endogenous reference. The qRT-PCR was performed using an ABI PRISM^®^ 7900HT system (Applied Biosystems, Foster, CA, USA).

### 4.6. Determination of Intracellular ROS Levels and Reduced Glutathione (GSH) Levels

To measure the levels of intracellular ROS, the cells were loaded with 10 μM of 2′,7′-dichlorofluorescein diacetate (DCF-DA) at 37 °C for 30 min. They were then washed 3 times with a phosphate buffered saline and their fluorescence intensities were analyzed using flow cytometry. The general antioxidant agent and ROS inhibitor *N*-acetyl cysteine (NAC, Sigma, St. Louis, MO, USA; 0–2 mM) was then added before further redox analysis. GSH Assay Kit (Abcam, Cambridge, UK) was used to measure glutathione concentrations as a marker of intracellular anti-oxidant capacity.

### 4.7. Flow Cytometry for Antibody Analysis

The anti-CD44-FITC (BD Biosciences, San Diego, CA, USA) antibody was analyzed to detect CD44 on the cell surface. PE-conjugated anti-N-cadherin (Abcam), FITC-conjugated anti-E-cadherin (Abcam) and xCT (Abcam) were also used to assess the cell surface antigens. The dissociated cells were double-stained with anti-human CD44 and anti-N-cadherin. The cells were then incubated at 4 °C for 15 min in the dark. Following incubation, the cells were washed once with a cold FACS buffer. The labeled cells identified by fluorescence intensity were analyzed using Gallious flow cytometry (Beckman Coulter, Brea, CA, USA), and the data were analyzed using FlowJo software (version 7.6.1; TreeStar: Ashland, OH, USA, 2012).

### 4.8. CD44 siRNA Knockdown

Small interfering RNA (siRNA) for CD44 (5′-GAACGAAUCCUGAAGACAUCU-3′, sense strand) was used. Cells were transfected with the siRNA by using Lipofectamine 2000 reagent (Invitrogen, Carlsbad, CA, USA), according to the manufacturer’s instructions. The cells were then incubated at 37 °C under 5% CO_2_ for 24 h.

### 4.9. Statistical Analysis

Data are expressed as mean ± SD from a minimum of 3 separate experiments. The differences between 2 groups were analyzed using the Student *t*-test. The differences among 3 groups were analyzed using one-way or 2-way ANOVA. A *p* value <0.05 was considered statistically significant. Statistical analyses were performed using SPSS for Windows version 14.0 (SPSS Inc.: Chicago, IL, USA, 2005).

## 5. Conclusions

The results of our study indicate the association between the functional CSC marker CD44 and the EMT phenotype, in correlation with redox status, in the CSCs of NPC. Our findings could potentially facilitate the development of an effective therapy for the prevention of NPC recurrence and metastasis.
